# Combination of Sorbitol and Glycerol, as Plasticizers, and Oxidized Starch Improves the Physicochemical Characteristics of Films for Food Preservation

**DOI:** 10.3390/polym13193356

**Published:** 2021-09-30

**Authors:** Berenice González-Torres, Miguel Ángel Robles-García, Melesio Gutiérrez-Lomelí, J. Jesús Padilla-Frausto, Claudia Luz Navarro-Villarruel, Carmen Lizette Del-Toro-Sánchez, Francisco Rodríguez-Félix, Arturo Barrera-Rodríguez, Mireya Zoila Reyna-Villela, María Guadalupe Avila-Novoa, Francisco Javier Reynoso-Marín

**Affiliations:** 1 Centro de Investigación en Biotecnología Microbiana y Alimentaria, Departamento de Ciencias Básicas, Centro Universitario de la Ciénega, Universidad de Guadalajara, Av. Universidad 1115, Ocotlán 47810, Mexico; berenice.gonzalez.220@estudiantes.ciad.mx (B.G.-T.); j.padilla@academicos.udg.mx (J.J.P.-F.); claudia.nvillarruel@academicos.udg.mx (C.L.N.-V.); avila.novoa@cuci.udg.mx (M.G.A.-N.); 2 Departamento de Investigación y Posgrado en Alimentos, Universidad de Sonora, Blvd. Luis Encinas y Rosales S/N, Hermosillo 83000, Mexico; carmen.deltoro@unison.mx (C.L.D.-T.-S.); francisco.rodriguezfelix@unison.mx (F.R.-F.); 3 Departamento de Ciencias Básicas, Centro Universitario de la Ciénega, Universidad de Guadalajara, Av. Universidad 1115, Ocotlán 47820, Mexico; arturo.barrera@academicos.udg.mx; 4 Departamento de Ciencias Tecnológicas, Centro Universitario de la Ciénega, Universidad de Guadalajara, Av. Universidad 1115, Ocotlán 47820, Mexico; mireya@cuci.udg.mx; 5 Departamento de Ingeniería en Nanotecnología, Universidad de la Ciénega del Estado de Michoacán de Ocampo (UCEMICH), Avenida Universidad 3000, Colonia Lomas de la Universidad, Sahuayo 59103, Mexico; fjreynoso@ucienegam.edu.mx

**Keywords:** plasticizers, glycerol (Gly), sorbitol (Sor), oxidized starch (OS), native starch (NS), films

## Abstract

The aim of this work was to use glycerol (Gly) and sorbitol (Sor) as plasticizers with oxidized starch potato (OS) to produce biodegradable and environmentally friendly films, and to demonstrate the resulting physicochemical and functional viability without subtracting the organoleptic characteristics of the food. Analyses by water vapor permeability (WVP), attenuated total reflection Fourier transform infrared spectra (ATR-FTIR), scanning electron microscopy (SEM), tensile strength (TS), and transparency (UV) showed that the best film result was with 1.5 g of Gly and 2.0 g of Sor, conferred shine, elasticity 19.42 ± 6.20%, and mechanical support. The starch oxidized to 2.5%, contributing a greater transparency of 0.33 ± 0.12 and solubility of 78.90 ± 0.94%, as well as less permeability to water vapor 6.22 ± 0.38 gmm^−2^ d^−1^ kPa^−1^. The films obtained provide an alternative for use in food due to their organic compounds, excellent visual presentation, and barrier characteristics that maintain their integrity and, therefore, their functionality.

## 1. Introduction

Ecological films have been studied to emphasize their alternative use with respect to traditional methods of food preservation, and to avoid petroleum-based compounds and pollution on land and sea, as these generate large amounts of solid waste and effects on health, in addition to their low cost and their easy and abundant acquisition [1]. Therefore, biodegradable films take part in the natural cycle of decomposition “from nature to nature” and play an important role in environmental sustainability; in addition, films play a key role in containing and protecting the nature of food from external factors such as oxygen, odors, microorganisms, and the integrity of its wall, among others. To improve the functionality of the films, combinations of polymers and, in turn, the integration of plasticizers have been investigated, to provide versatility in the mechanical properties and gas/moisture barrier properties depending on interactions between components within the film structures [2,3,4].

Starch is a polysaccharide that is divided into two macromolecules: 20–30% amylose and 70–80% amylopectin, a content that depends on the origin of the starch. It is one of the most abundant organic compounds in nature in that it is found in roots, tubers, fruits, and grains [5]. Amylose is a linear glucose polysaccharide linked to α-1-4 glucosidic bonds that provides the molecule with a semicrystalline form, while amylopectin is a branched polysaccharide with α-1-6 bonds and is associated with the amorphous region [6].

Native starch (NS) is one of the most widely used components due to its easy acquisition and low cost, and the advantages of its industrial use in the food, pharmaceutical, paper, and textile industries; among the characteristics that stand out are its properties as an emulsifier, humectant, thickener, controlled-release system controller, and film former. NS, when modified by an oxidizing agent, provides better physical, functional, and structural characteristics according to the specific requirements of use [7,8]. In addition, temperature, time, and pH are controlled during the oxidation process with the oxidizing agent, as they will determine the success of the oxidation, the future reactions that arise in the process, the final form of the OS, and its characteristics. OS provides desirable physical–mechanical properties, including better hydration and swelling of the molecule, low viscosity, better transparency, and greater stability compared with a NS [9,10]. Higher oxidation of NS with the oxidizing agent results in a higher crystallinity peak also reflected in an increase in water vapor transference in the films, which is due to the interactions of the polymer chains, and between the polymer chains and the plasticizers [11].

An edible film comprises a thin, polymeric matrix of edible materials, on which all of the determination and physicochemical characterization tests are carried out to be placed at a later time on the surface of food without subtracting its organoleptic properties [12]. Plasticizers such as Gly and Sor, known as polyols, are commonly used additives and are most popular partly due to their low cost, but primarily because they provide flexibility characteristics to the films by reducing hydrogen bonds in the polymer chains, increasing the molecular space—in turn, adding a better visual appearance [8,13]. Glycerol (Gly) is an extremely small organic molecule that can enter into the starch molecules; Sorbitol (Sor) is a small molecule that is resistant to water for the reason that is has less affinity to water and a stronger molecular interaction with polymer chains [2]. Both optimize the desired characteristics of the film; an example of this lies in avoiding fractures during handling and a film with a useful life as a coating, giving it shine and flexibility. The amount of each of these ingredients influences in such a way that Gly possesses characteristics such as the long transmission of vapor to water, high solubility, greater elongation, and low tensile strength, while Sor contributes to shine, less vapor transmission of water, and greater mechanical support [8].

In this context, the objective of the study consisted of characterizing the films with Gly, Sor, and OS added, for their use in coating some foods and determining the advantage and potential effects of using both plasticizers, as well as the advantages in the film when using an oxidized starch. The films were characterized to describe their morphological, barrier, and molecular-interaction properties, as well as their mechanical properties and future implementation.

## 2. Materials and Methods

### 2.1. Materials

The materials include unmodified native Potato Starch (PS) (Hycel), sodium hypochlorite (NaOCl) containing 5.1% active chlorine, glycerol, sorbitol (Sigma-Aldrich, San Luis, MO, USA), and other reagents of analytical grade. 

### 2.2. Preparation of Oxidized Starches

A starch suspension was prepared according to Wang and Wang [10] with some modifications in the concentrations of NaOCl utilized for the oxidation of starch (2.0, 2.5, 3.0, 3.5, 4.0, and 4.5% active chlorine (*w*/*v*). The starch suspension was maintained at 35 °C for 30 min and the pH was adjusted to 9.5 with 2 N NaOH. The NaOCl was slowly added to the starch slurry over 30 min while maintaining the pH at 9.5 with 1 N H_2_SO_4_. After the addition of the NaOCl solution, the pH of the slurry was maintained at 9.5 with 1 N NaOH for an additional 50 min. The slurry was then adjusted to 7.0 with 1 N H_2_SO_4_ and filtered by suction with a Buchner filter funnel (Whatman filter #4), washed with several volumes of deionized water, and dried in a convection oven at 40 °C for 48 h; after some time, it was ground with a mortar.

### 2.3. Carbonyl Group Content

The carbonyl group content in OS was determined following the titrimetric method of Smith [14] and calculated as follows:

(1) $$\%\mathrm{Carbonyl}\;\mathrm{group}\;\mathrm{content}\;=\frac{\left[\left(\mathrm{Blank}-\mathrm{Sample}\right)\;\mathrm{mL}\;\times \mathrm{Acid}\;\mathrm{normality}\;\times 0.028\times 100\right]}{\mathrm{Sample}\;\mathrm{weight}\;\left(\mathrm{dry}\;\mathrm{basis}\right)\;\mathrm{in}\;g}$$

### 2.4. Carboxyl Group Content

The carboxyl group content of OS was determined according to the modified procedure of Chattopadhyay, Singhal, and Kulkarni [15] and the carboxyl content was calculated as follows:

(2) $$\frac{\mathrm{milliequivalents}\;\mathrm{of}\;\mathrm{acidity}\;}{100\;g\;\mathrm{starch}}=\frac{\left[\left(\mathrm{Sample}-\mathrm{Blank}\right)\;\mathrm{mL}\times \mathrm{Normality}\;\mathrm{of}\;\mathrm{NaOH}\;\times 100\right]}{\;\mathrm{Sample}\;\mathrm{weight}\;\left(\mathrm{dry}\;\mathrm{basis}\right)\;\mathrm{in}\;g}.$$

(3) $$\%\mathrm{Carboxyl}\;\mathrm{group}\;\mathrm{content}\;=\left[\frac{\mathrm{milliequivalents}\;\mathrm{of}\;\mathrm{acidity}}{100\;g\;\mathrm{starch}}\right]\times 0.045$$

### 2.5. Film Preparation 

Eight film formulations were prepared using 2.0 g of OS in all, and the study factors included Sor, Gly, and OS with their respective levels—Sor (2.0 and 2.5 g), with Gly (1.0 and 1.5 g), OS (2.0 and 2.5%), and distilled water to complete 100 g of solution. The concentration of the compounds was based on preliminary tests and the films with OS at extensities of modification of 3.0, 3.5, 4.0, and 4.5% were discarded because they did not form.

The solution contained a mixture of all of the compounds and was heated at 95 °C and 125 rpm for 15 min. Then, according to the casting technique, 20 mL for each filmogenic solution was poured into a Petri dish with a diameter of 9 cm to obtain a constant film thickness (0.15 ± 0.01 mm) regardless of the composition and was dried in an oven at 40 °C for 48 h. Dry films were peeled off and stored at 57 ± 1% RH and 25 °C for 4 days before any testing.

### 2.6. Scanning Electron Microscopy (SEM)

The surface homogenization of the films and the morphology of the dry starches were visualized by scanning electron microscope (JSM-6610LV; Peabody, MA, USA) with an accelerating voltage of 10 kV. Samples were then placed in a stub with carbon tape, coated with gold using a Desk V sputter (Moorestown, NJ, USA), and examined using magnifications of 200× and 1000×.

### 2.7. Thickness and Mechanical Properties of the Films

Film thickness was measured using a micrometer (Model Truper 14388; Tepotzotlán, Mexico) to the nearest 0.05 mm at 6 random positions around the film, where average rates were employed in the calculations. Tensile strength (TS, MPa) and the percent of elongation at the break (E) (%) were evaluated by a tensile test performed on a texture analyzer (TA, ElectroForce, BioDynamic 5100; New Castle, DE, USA) according to ASTM-D1708 [16]. Filmstrips (25 mm × 10 mm) were cut from each preconditioned sample and mounted between the grips of the equipment. The initial distance between the grips and the initial velocity were adjusted to 4 mm and 0.1 mm/s, respectively. Mechanical properties were calculated using the average thickness of each film sample and replicated 5 times. 

### 2.8. Moisture, Solubility in Water, and Water Vapor Permeability (WVP) of the Films

The moisture content was determined following AACC-44-15 [17], with modifications, measuring the weight loss of the 4 cm-diameter film circle, after drying it in an oven at 100 °C for 8 h until the weight was constant. The results were expressed with the following equation:

(4) $$\%\mathrm{Moisture}\;=\frac{\mathrm{lost}\;\mathrm{weight}\;\left(g\right)}{\;\mathrm{initial}\;\mathrm{weight}\;\left(g\right)}$$

Solubility in water (%SW) was determined using a 4 cm-diameter dry film circle (Po) that was placed in 50 mL distilled water at 25 °C for 24 h with occasional agitation. The solution was filtered and the undissolved film was dried at 100 °C for 24 h (Pf). The weight of the solubilized dry matter was calculated according to El Halal et al. [18] by the following equation:

(5) $$\%\mathrm{SW}=\frac{\mathrm{Po}-\mathrm{Pf}\;}{\;\mathrm{Po}}$$

WVP tests of the films were performed following the ASTM E96-E96M [19] standard method. Each sample was placed onto a circular test opening of a permeation cell containing anhydrous calcium chloride (0% RH). Then, the samples were placed in a chamber at 45% RH and maintained at 25 °C. Weight changes were recorded daily for 5 days (0, 12, 24, 36, 48, 72, 96, and 120 h). WVP was calculated with the following equation:

(6) $$\mathrm{WVP}=\left(\;\frac{\Delta W}{\;t}\right)\times \left(\;\frac{X}{\;A\Delta P}\right)$$

where Δ*W* is the gained weight in the chamber (g), *t* is the time, *X* is the thickness (mm), *A* is the exposed area (m^2^), and Δ*P* is the partial pressure difference. Three replicates were tested for each sample.

### 2.9. Light Transmittance and Transparency

Light transmittance was determined according to Murrieta-Martínez et al. [20] with modifications. Films were cut into strips (1 cm × 4 cm) and placed on the inside wall of a quartz cuvette (1 cm). The ultraviolet (UV) and the visible light barrier of the films were measured at between 350 and 800 nm. Transparency was calculated employing the following equation:

Transparency = *A*_600_/x (7)

where *A*_600_ is the absorbance at 600 nm and x is the film thickness (mm).

### 2.10. Attenuated Total Reflection Fourier Transform Infrared (ATR-FTIR) Spectroscopy

Films, NS, and dry OS were characterized and we showed the interactions of functional groups due to plasticizer and the different levels of extensity of modification in the starch using an FTIR spectrometer (Shimadzu, Kyoto, Japan) equipped with an attenuated total reflection (ATR) accessory, measured directly onto a solid-state sample surface by pressing the sample towards a multicrystal ATR crystal, in a range between 4000–500 cm^−1^ with a resolution of 4 cm^−1^.

### 2.11. X-ray Diffraction 

The starches were analyzed with an X-ray diffractometer (Theta-Theta, Darmastadt, Hesse, Germany) using CuKα radiation (λ = 0.154 nm), 35 Kv, and 30 mA with a 2θ scan region ranging from 5° to 40° and a 60 s count time per angular step. The material was placed on a 30-mm × 30-mm aluminum sample. Before the measurement, the samples were subjected to a drying process at 40 °C for 48 h to avoid measuring them with humidity.

### 2.12. Statistical Analysis

All the experiments were performed in triplicate, and the data were evaluated using an analysis of variance (ANOVA), followed by a Fisher’s least significant difference (LDS) test, using the Statgraphics Centurion XVI software (StatPoint Technologies, Inc., Warrenton, VA, USA). Data were presented as mean ± standard deviation (SD). 

## 3. Results and Discussion

### 3.1. Carbonyl and Carboxyl Contents

The carbonyl and carboxyl contents in the OS are listed in Table 1. During the oxidation reaction, the hydroxyl groups are oxidized first into carbonyl and then into carboxyl groups; the concentration of carbonyl groups and carboxyl groups in starch will be a function of the concentration of NaClO used for the oxidation reaction [10]. The obtained results differed from those of other authors [10,21], reflecting that there is a higher percentage of carbonyl groups than of carboxyl; this is because the oxidizing agent was probably not as strong, still causing the reaction of hydroxyl groups to carbonyls, but not so high that a higher concentration of carboxyl groups was formed, regardless of the concentrations of the pH value employed during oxidation. Zhou et al. [22] suggested that in high oxidation, the number of carboxyl groups will determine the level or degree of oxidation. The content carbonyl groups with 2.0 and 2.5% of NaClO were not significantly different (*p* > 0.05). However, it is appreciated that the carboxyl group increases when NaClO increases. These were statistically different (*p* < 0.05), which is why the films possess better characteristics in the first two extensities of modification.

### 3.2. Films Obtained 

**Figure 1:**
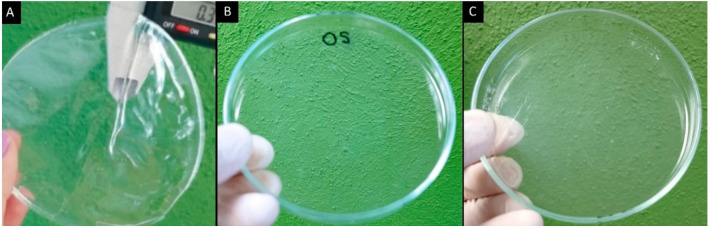
Developed films: (**A**) formulation 6 (plasticizers and OS), (**B**) OS without plasticizers, and (**C**) NS, prepared using the solution casting method.

Films were formulated with the OS from 2.0 to 4.5% to evaluate their characteristics. However, the films formulated with extensities of modification of 3.0, 3.5, 4.0, and 4.5% did not yield the desired characteristics and, as a result, were opaque, rigid, and brittle. This is because at a higher extensity of modification, the molecule of the starch is deformed, and due to the degradation of the crystalline lamellae, the carbonyl and carboxyl groups introduced into the starch molecule promote swelling of the granules. When the solution prior to the casting technique is heated it has a lower viscosity than when the oxidation concentration increases; however, when the solution is cooled, it has a viscous appearance [9,10]. Instead, the concentrations of 2.0 and 2.5% were transparent and flexible, while the films without plasticizers were opaque and very rigid—they could not even be detached from the Petri dish due to the lack of plasticity. The use of both plasticizers in the same formulation provides a better characteristic: Gly provides flexibility to the film and Sor confers rigidity and brightness [20,23]. No cracks on the film surface were observed in Figure 1A. 

### 3.3. Scanning Electron Microscopy (SEM)

**Figure 2:**
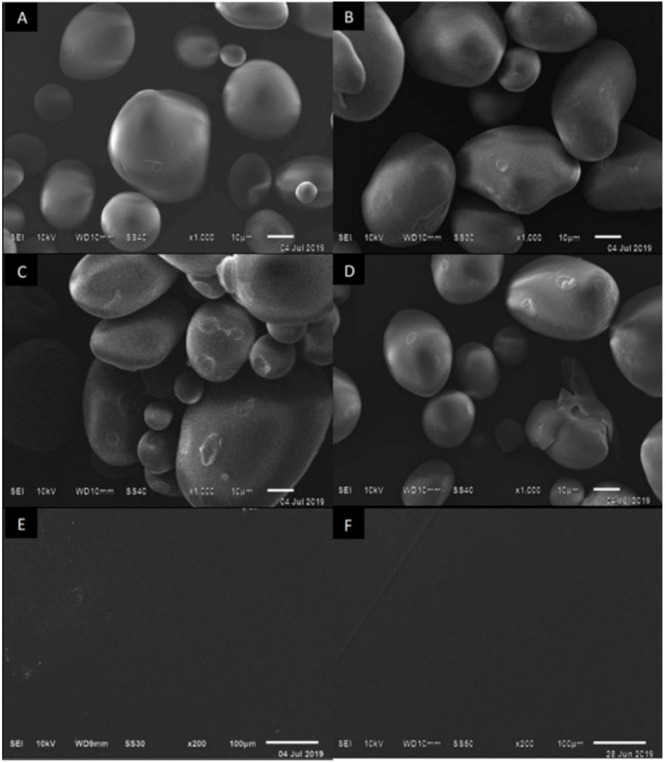
SEM micrographs of the surfaces. (**A**) Native starch and oxidized starches at (**B**) 2.0%, (**C**) 2.5%, (**D**) 3.0%. (**E**) Films with 2.0% of OS and plasticizers. (**F**) Films with 2.5% of OS and plasticizers. Magnitudes are 1000× and 200×.

With the scanning electron microscopy technique, the external structure of the NS was observed, which had a smooth wall, without defects, and different sizes of ovoid and circular appearance (Figure 2A), as reported by Velásquez-Herrera et al. [24]. As the extensity of modification increased, the OS presented fissures and pores as well as deformation of the granule in its crystalline structure (Figure 2B–D). This coincides with the work carried out by Kuakpetoon and Wang [21], Zhou et al. [22], and Vanier et al. [25], which mention that greater extensity of modification will bring about a deformation of the granule and instability in the solution for the formation of the film. Therefore, the change in the morphology of OS—such as the amylose quantity, molecular structure, and its size and shape—depends on the reaction conditions in oxidation including pH, temperature, time, starch source, NaClO concentration, and the characteristics of the starch [25].

The surface morphology of the films at extensities of modification of 2.0 and 2.5% (Figure 2E,F) exhibited good homogeneity and excellent acceptance of Gly and Sor, without pores or cracks, which is acceptable for controlled permeability and the gas exchange of the film. When OS and plasticizers interact, such as carbohydrates that have the same polarity, strong interaction bonds are generated, which make the appearance of the film clear and homogeneous [2].

### 3.4. Thickness and Mechanical Properties of the Films

In terms of the film’s thickness, there is a general trend toward formulations prepared from 0.14 to 0.18 mm, due to the volume utilized for the formation of the film and the presence of dry solids after the drying process (Table 2). This parameter will determine the transfer of gases in the respiration of the product and the water vapor, transparency, mechanical properties, and initial organoleptic characteristics.

For the film to be functional, it must possess resistance, stability, and flexibility, and must withstand temperature and physical changes when incorporated into a food, despite the stress conditions throughout the chain, until it reaches the consumer [26]. Tensile strength (T), the percentage of elongation at the breaking point (%E), and Young’s modulus (Y) are displayed in Table 2. These depend on the quantity and type of components employed as follows: (1) the extensity of modification, (2) the plasticizers, and (3) water [11]. Both plasticizers confer on films a reduced behavior of tensile strength < 0.57 ± 0.12 MPa, rendering the polymer matrix less dense, facilitating the movement of polymer chains, granting flexibility or ductility, and forming a hydrophilic separation in conjunction with the starch and the water [26,27].

As described by Zamudio-Flores et al. [11], hydrogen bridge bonds between the carbonyl and carboxyl groups can occur with OH- groups from amylose and amylopectin molecules. This is to provide greater integrity of the polymer matrix; therefore, a higher extensity of modification at 2.5% provides greater tensile strength and a lower amount of Gly (1 g), while Sor does not show a significant difference in the quantities evaluated (2.0 and 2.5 g). Nevertheless, the elongation at break did not show a significant difference in OS (2.0 and 2.5%) and Gly (1.0 and 1.5 g), while Sor at 2.5 g had a higher elongation at break. During the film’s formation, the high temperature destroyed the crystallinity structures and the resulting structures exist only in an amorphous phase [8]. According to the results obtained, the extensity of modification in the OS and the amount of Gly significantly influence the effort required to reach the breaking point of the biofilm. Nonetheless, if the amount of plasticizer is increased, this would decrease. Talja et al. [28] and Davoodi et al. [29] observed that the combination of both plasticizers—that is, Gly and Sor—decreased resistance to tension, which agrees with the obtained results. The use of both of these plasticizers reduces the intramolecular affinity in the starch, forming new hydrogen bonds with the plasticizers, conferring greater flexibility, and avoiding breakage. In another study, Laohakunjit and Noomhorm [30] reported that Gly and Sor entertain differences in the film when employed separately in a rice starch film, and Gly possesses lower tension than Sor with a value of 5.41 MPa; however, Gly has greater elongation. The changes in Young’s modulus were identified for the increase of both plasticizers Gly 1.5 g and Sor at 2.5 g—that is, the greater the amount (in g) of plasticizers, the greater the longitudinal elasticity; however, the values are < 0.73 ± 0.20 MPa. The films in this study are influenced by the use of both, where it shows a tension of less than 1 MPa and less formation of hydrogen bridges is demonstrated, due to the low amount of carbonyl and carboxyl groups in the extensity of modification, but with a percentage of deformation greater than 19.42 ± 6.20.

The conditions of the film will depend not only on its chemical structure, but also on external factors such as the RH gradient and the type of surface where it is placed, among others, and it is required to withstand damage during its food processing.

### 3.5. Moisture, Solubility in Water, and WVP of the Films

The less amount of moisture the film has, the better the performance in preventing the growth of microorganisms [31], but there must be a balance; films with zero or very low moisture cannot be made because this would have an impact on quality characteristics such as color and brittle texture, leaving the food surface exposed. The results are presented in Table 3. Films made with OS at 2.5%, 2 Sor, and 1.5 of Gly had lower than 52.86% of moisture but this was high compared to that obtained by Hazrol et al. [2] who incorporated both. Hu et al. [8], when increasing the amounts of Gly, found that hydroxyl groups in glycerol have a strong affinity with water molecules so the moisture increases. However, the films have a high-water retention capacity, which benefits their structure; the opposite happens with the Sor, the molecular similarity of starch and sorbitol contribute to its affinity and interaction to water and have a stronger molecular interaction with the polymer chains [32,33].

The results of solubility and permeability are presented in Table 3. The variation in the number of plasticizers employed, as well as the oxidation, do not exhibit a significant difference in solubility (Table 3). However, the use of both plasticizers and their preparation with OS by heat gelatinization renders them more soluble when they come into contact with water [34], noting that solubility should be taken into account for the food to be applied. The values obtained are high if a rapid disintegration is desired, compared with those obtained by Zavareze-Eda et al. [35], who evaluated the solubility of OS films and reported values ranging from 14.26% to 19.89%. WVP is a property that is of great importance, since the lower transfer of moisture that the food may have, is ideal for maintaining the food fresh for a long time, and without microbial growth that deteriorates the quality of the food inside [32]. Due to this, the film must a lower WVP; otherwise, it would be a poor barrier, according to Zavareze-Eda et al. [35]. El Halal et al. [18] reported that oxidation promotes the repulsive forces among the polymer chains, opening spaces that give rise to the water vapor migrating through the coating. In this study, the statistical analysis revealed that an OS of 2.5% has a higher WVP than OS at 2.0% (Table 3). Gly entertains significance in WVP, since it possesses an affinity to water, promotes the diffusion of molecules, and its relatively small size makes it easy for it to insert itself between amylose and amylopectin molecules in starch, establishing hydrogen bonds with the carboxyl groups. A similar tendency has been reported for Souza et al. [36] for increasing the concentration of Gly; the WVP increases, while Sor has the ability to maintain a low moisture content due to its long polarity [37]. The ability to retain water will be influenced to a greater degree by hygroscopic plasticizers than by starch [38].

### 3.6. Light Transmittance and Transparency of the Films

The transparency of a film is of great importance since it reflects its presentation when incorporated on the surface of food. It is said that the cover must be transparent to the human eye—that is, 90% (600 nm) [20]. The results of the films obtained with the combination of Gly and Sor are presented in Table 4. The readings from 350 to 800 nm were for determining whether the components intervened in the transmittance; in previous investigations, each component utilized separately presents different values in the different ranges of length. Nonetheless, there is no significant difference when the components are used together and in any composition. With respect to transparency, this value must be as close as possible to 1; thus, coinciding with a study carried out by Blanco-Pascual et al. [39], where the authors observed that when using Gly and Sor, they obtained a transparency of 0.6; Murrieta-Martínez et al. [20] obtained a Gly transparency of 0.5 and Sor transparency of 0.7 when employing them separately, demonstrating that Sor provides greater transparency in films. Both characteristics will be influenced by the thickness of the coating, which is due to the amount by weight of the materials utilized: the greater the quantity, the greater the opacity, and the greater its chemical composition [40]. 

### 3.7. Attenuated Total Reflection Fourier Transform Infrared (ATR-FTIR) Spectroscopy

**Figure 3:**
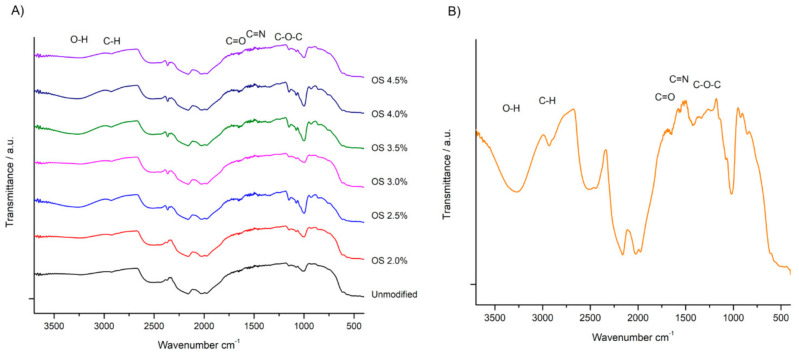
(**A**) ATR-FTIR spectra of native starch and oxidized starch with different degrees of substitution and (**B**) spectra of the film with plasticizers, formulation 6.

ATR-FTIR spectra are useful for observing and understanding the molecular interactions that starches engage in at their different extensities of modification (Figure 3A) and the OS 2.5% film with all of their components (Figure 3B). The regions at 3600 and 3100 cm^−1^ denote broad bands with –OH junctions, with a hydrophilic tendency that, when the Gly plasticizer is present in the films, is more intensified; therefore, they contain a higher amount of hydroxyl groups than starch and hydrogen bonding [41]. A region at 2920 cm^−1^ attributed to the stretching vibration of C-H bonds and a region at 1700 and 1630 cm^−1^ weakly reveal the presence of C=O bond [42], while the band in the 1620 cm^−1^ region similarly presents a weak intensity, related to the C=N group, this is due to the dialdehyde polysaccharides capable of crosslinking with the amino group’s protein. In the 1600 cm^−1^ band, the bending of bonds is closely observed in water, and in the 1150 and 1050 cm^−1^ regions, vibrations of the glycosidic bonds such as pyranose C-O-C are involved due to the breakdown of glucose at the time of degradation. The first visible peak is in the 1000 cm^−1^ region, with a C-O link extension [43]. Although this is the same molecule, there are slight changes due to the added NaOCl that change its initial conformation. While the film absorption levels are shown in the same regions and the presence of polyols, regardless of the type and concentration of the plasticizer [32].

### 3.8. X-ray Diffraction

**Figure 4:**
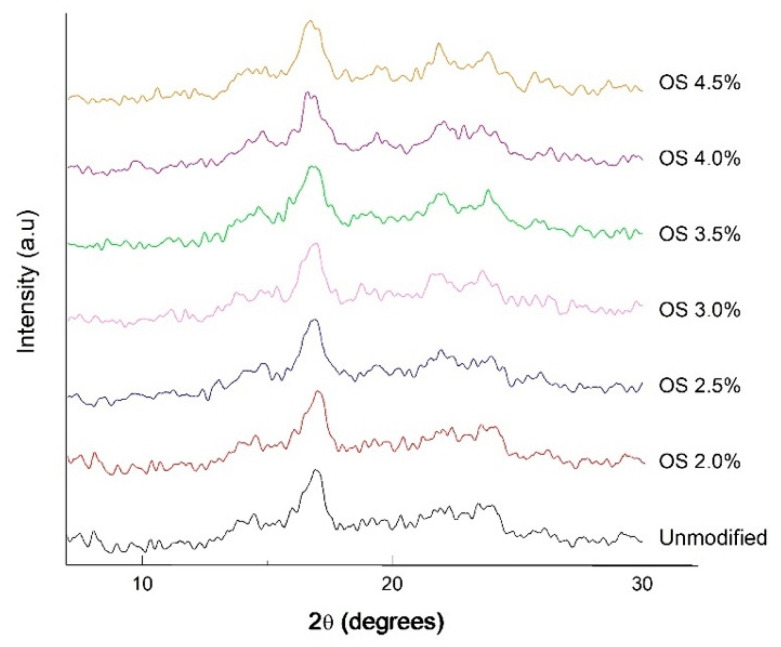
X-ray diffractograms of native starch and oxidized starch with different degrees of substitution.

The X-ray diffraction patterns in Figure 4 show that NS has characteristic peaks at 2ϴ = 14°, 2ϴ = 17°, 2ϴ = 19°, 2ϴ = 21°, and 2ϴ = 23° corresponding to a typical semicrystalline solid structure with B-type crystallinity typical for tuber starches. This structure can be interpreted as having hexagonal symmetry due to amylopectin-hydrated double helix packing [44]. OS at different extensity of modification also presents a semicrystalline structure, with similar peaks at 2ϴ = 14°, 2ϴ = 17°, 2ϴ = 19°, 2ϴ = 22°, and 2ϴ = 24°. The diffraction peak at 2ϴ = 18° is in good agreement with Zobel [45], who reported that B-type crystallinity has a peak at 2ϴ = 18°, whereas the peak at 2ϴ = 21° is related to the presence of complexes between the amylose and the lipids [46]. On the other hand, according to Rivas-González et al. [47], the chemical modifications do not affect the diffraction pattern, but instead the percentage of crystallinity of the starch, since extensity of modification tends to take place first in the amorphous phase and last in the crystalline phase. It should be noted that the degree of crystallinity is directly related to the amount of amylopectin, while the amorphous phase is related to the amylose that this polymer contains. The importance of characterizing starch is based directly on its possible application for the food industry.

## 4. Conclusions

The film was successfully fabricated utilizing Gly and Sor with OS using the casting technique. Due to the homogeneous dispersion of plasticizers with OS, there is an improvement in the properties of the film. Based on mechanical barrier tests, plasticizers Gly and Sor increase the versatility of films, revealing a decrease in tensile strength preventing breakage and good elasticity. These characteristics are desirable to maintain the integrity of the food until it reaches the consumer’s hands, in addition to being a biodegradable and sustainable film due to the components used. 

Micrographs by SEM exhibited good acceptance in the film, showing smooth surfaces and an excellent homogeneous matrix without cracks or pores when observed. The incorporation of both plasticizers with OS also revealed good transparency; greater solubility by the water affinity of the components, if this is desired for its application in fast-food products; and lower WVP, desirable for less weight loss of the food and preservation of their organoleptic properties. Evaluation of the physicochemical characteristics at the molecular level with ATR-FTIR and XRD analyses allowed us to observe changes in the structure of the starch as well as the bonds formed. Depending on the desired industrial application and the food to be tested, packaging requires different properties. The presented results are preliminary but can contribute to emphasize the applicability of the discussed method at a large scale. More studies are needed to evaluate the behavior of films in the food matrix.

## Figures and Tables

**Table 1:** The carbonyl and carboxyl group content of OS with different concentrations of extensity of modification.

%NaClO	Carbonyl Group Content (%)	Carboxyl Group Content (%)
2.0	0.188 ± 0.004 ^a^	0.00052 ± 0.00002 ^e^
2.5	0.188 ± 0.004 ^a^	0.00074 ± 0.00001 ^d^
3.0	0.131 ± 0.004 ^ab^	0.00076 ± 0.00001 ^d^
3.5	0.174 ± 0.004 ^bc^	0.00140 ± 0.00002 ^c^
4.0	0.171 ± 0.004 ^c^	0.00163 ± 0.00001 ^b^
4.5	0.176 ± 0.007 ^bc^	0.00170 ± 0.00001 ^a^

Different letters indicate a significant difference (*p* < 0.05) in the content of carbonyl and carboxyl group. Values are the means of three replicates ± standard deviation.

**Table 2:** Composition, thickness, and mechanical properties of the films.

Films Composition		Thickness (mm)	Tensile Strength (MPa)	Elongation at Break (%)	Young’s Modulus (MPa)
NaClO (%)	Plasticizers (g)				
2.0	(1) 2 Sor-1 Gly	0.16 ± 0.06 ^aaa^	0.57 ± 0.12 ^baa^	21.14 ± 4.19 ^aba^	0.26 ± 0.04 ^abb^
	(2) 2 Sor-1.5 Gly	0.14 ± 0.03 ^aaa^	0.25 ± 0.02 ^bab^	22.44 ± 7.27 ^aba^	0.60 ± 0.09 ^aba^
	(3) 2.5 Sor-1 Gly	0.14 ± 0.05 ^aaa^	0.34 ± 0.06 ^baa^	35.31 ± 12.22 ^aaa^	0.60 ± 0.16 ^aab^
	(4) 2.5 Sor-1.5 Gly	0.15 ± 0.04 ^aaa^	0.23 ± 0.11 ^bab^	21.02 ± 6.91 ^aaa^	0.48 ± 0.15 ^aaa^
2.5	(5) 2 Sor-1 Gly	0.15 ± 0.08 ^aaa^	0.49 ± 0.17 ^aaa^	19.43 ± 6.63 ^aba^	0.29 ± 0.09 ^abb^
	(6) 2 Sor-1.5 Gly	0.16 ± 0.07 ^aaa^	0.36 ± 0.13 ^aab^	19.42 ± 6.20 ^aba^	0.39 ± 0.11 ^aba^
	(7) 2.5 Sor-1 Gly	0.14 ± 0.05 ^aaa^	0.47 ± 0.09 ^aaa^	30.00 ± 10.61 ^aaa^	0.45 ± 0.12 ^aab^
	(8) 2.5 Sor-1.5 Gly	0.18 ± 0.07 ^aaa^	0.39 ± 0.10 ^aab^	40.46 ± 4.96 ^aaa^	0.73 ± 0.20 ^aaa^

Different letters indicate a significant difference (*p* < 0.05) between the levels of a factor. The first letter corresponds to the OS factor, the second to Sor, and the third to Gly, which will depend on the value required for each test.

**Table 3:** Moisture, water solubility, and water vapor permeability (WVP) of the films.

Film	%Moisture	%Solubility	$\mathrm{WVP}\;\left[\frac{g\cdot mm}{m^{2}\cdot d\cdot Kpa}\right]$
1	74.55 ± 2.61 ^bab^	74.05 ± 3.13 ^aaa^	3.52 ± 0.29 ^aaa^
2	69.95 ± 3.00 ^baa^	75.21 ± 0.59 ^aaa^	2.64 ± 0.18 ^aab^
3	77.26 ± 7.32 ^bbb^	74.95 ± 1.22 ^aaa^	3.56 ± 0.21 ^aaa^
4	67.87 ± 0.91 ^bba^	76.64 ± 4.31 ^aaa^	3.19 ± 0.14 ^aab^
5	73.60 ± 2.13 ^aab^	73.23 ± 3.36 ^aaa^	3.05 ± 0.42 ^baa^
6	52.86 ± 2.36 ^aaa^	78.90 ± 0.94 ^aaa^	6.22 ± 0.38 ^bab^
7	73.04 ± 4.51 ^abb^	76.21 ± 0.77 ^aaa^	4.27 ± 0.68 ^baa^
8	70.66 ± 4.76 ^aba^	76.10 ± 5.46 ^aaa^	0.27 ^bab^

Different letters indicate a significant difference (*p* < 0.05) between the levels of a factor. The first letter corresponds to the OS factor, the second to Sor, and the third to Gly, which will depend on the value required for each test.

**Table 4:** Transmittance and transparency of films.

Film	Light Transmittance (%)						Transparency
	350	400	500	600	700	800	
1	62.77 ± 1.18	86.17 ± 0.23	88.11 ± 0.20	88.51 ± 0.00	89.33 ± 0.21	89.40 ± 0.24	0.27 ± 0.09 ^aaa^
2	61.10 ± 1.12	84.99 ± 1.53	86.84 ± 1.41	87.30 ± 1.41	87.98 ± 1.32	88.31 ± 1.13	0.33 ± 0.03 ^aaa^
3	63.22 ± 2.17	86.57 ± 1.09	88.38 ± 0.77	88.72 ± 0.74	89.47 ± 0.66	89.61 ± 0.63	0.29 ± 0.08 ^aaa^
4	62.23 ± 0.38	86.03 ± 0.30	87.84 ± 0.51	88.24 ± 0.62	88.85 ± 0.63	89.06 ± 0.63	0.34 ± 0.10 ^aaa^
5	64.04 ± 1.69	86.83 ± 0.12	88.65 ± 0.12	89.13 ± 0.21	89.74 ± 0.21	89.88 ± 0.32	0.38 ± 0.21 ^aaa^
6	62.57 ± 1.23	85.97 ± 1.02	87.84 ± 0.77	88.24 ± 0.59	88.85 ± 0.59	89.06 ± 0.59	0.33 ± 0.12 ^aaa^
7	63.15 ± 0.95	86.70 ± 0.80	88.45 ± 0.85	88.72 ± 0.74	89.47 ± 0.86	89.61 ± 0.83	0.42 ± 0.15 ^aaa^
8	63.41 ± 1.95	85.51 ± 0.90	87.23 ± 0.70	87.63 ± 0.71	88.24 ± 0.71	88.45 ± 0.71	0.32 ± 0.12 ^aaa^

The analysis reveals that, for the transparency measured at 600 nm, there is no significant difference with the 95% confidence interval by LSD. The first letter corresponds to the OS factor, the second to Sor, and the third to Gly, which will depend on the value required for each test.

## Data Availability

Not applicable.
